# Stress adaptation signature into the functional units of spike, envelope, membrane protein and ssRNA of SARS-CoV-2

**DOI:** 10.22099/mbrc.2022.44594.1777

**Published:** 2022

**Authors:** Aniket Sarkar, Anindya Sundar Panja

**Affiliations:** 1 Post Graduate Department of Biotechnology, Oriental Institute of Science and Technology, Vidyasagar University, Midnapore, 721102, West Bengal, India; 2 Post-Graduate Department of Biotechnology, Molecular informatics Laboratory, Oriental Institute of Science and Technology, Vidyasagar University, Midnapore, West Bengal, India

**Keywords:** SARS-CoV-2, Spike, Membrane, Envelope, Stress, Stability Pocket

## Abstract

Pandemic coronavirus causes respiratory, enteric and sometimes neurological diseases. Proteome data of individual coronavirus strains were already reported. Here we investigated of SARS-CoV-2 ssRNA and protein of spike, envelope and membrane to determine stress adaptation profile. Thermodynamic properties, Physicochemical behaviour and, amino acid composition along with their RMSD value was analysed. Thermodynamic index of SARS-CoV2 spike, envelope and membrane ssRNA is unstable in higher temperature. Presence of higher proportion of polar with positive and negative charged amino acid residues into spike (S), envelope (E) and membrane (M) protein indicate the lower stress adaptability pattern. Our study represented several unstable pockets into S, E and M proteins of SARS-CoV-2 against different abiotic stresses, specifically higher in spike protein. Contact with heat through solvent may denature the architectural network of SARS-CoV-2 spike, envelope and membrane ssRNA and structural protein. The stress instability index of SARS-CoV-2 and the interactome profile of its transmembrane proteins may help to reveal novel factors for inhibiting SARS-CoV-2 growth.

## INTRODUCTION

Zoonotic beta coronaviruses can lead to severe acute respiratory syndrome or mild pneumonia [1]. Coronavirus genome consists of single-stranded positive-sense RNA which codes for 26 different proteins [2]. This virus has the potential to rapidly mutate and spread throughout the human community [3, 4]. Understanding the function of spike, envelope, and membrane ssRNA in biological sciences necessitates the disclosure of their thermodynamic properties. In the presence of water, spike, envelope, and membrane ssRNA may disintegrate quickly, but heat or enzymes prevent this from happening. This variation in degradation rates is critical for understanding how spike, envelope and membrane ssRNA influences biological processes. Numerous aspects of the life cycles of various viral species have already been subjected to thermodynamic analysis. The biology of the viral life cycle and the procedures employed to create spherical virus capsids are both newly illuminated by the thermodynamics of capsid assembly for a variety of viruses, which approaches it as a polymerization event [5]. 

Coronavirus structural proteins include the spike (S)protein, envelope (E)protein, membrane (M)protein, and nucleocapsid (N)protein [6, 7]. The four coronavirus genera' entry into host cells is strictly controlled by the envelope-anchored spike protein [8]. The pre-fusion homo-trimer structure of the spike protein contains three receptor-binding S1 heads adhering with trimeric membrane-fusion S2 stalk [9, 10]. The Extracellular domain of spike protein contains 14–1,195 amino acids, transmembrane domain (TM) 1,196–1,216 amino acids and a cytoplasmic domain (CP) contains the rest of the residues. The S protein contains two chains, subunits S1 three domains (I, II, and III) and S2 with two helix regions (HR1 and HR2) [11, 12]. The S1 subunit of the SARS-CoV-2 spike protein binds to the cellular receptor angiotensin converting enzyme 2 (ACE2) and S2 subunit to the type II transmembrane serine protease TMPRSS2 which initiate the activation of host cell protease [13-15]. The complex of host cell protease TMPRSS2 and S2 subunits of SARS-CoV-2 is located to the cleavage site and proteolysis is done by three amino acids, positioning 795–797 [16, 17]. Trans-membrane (M) and envelope (E) are both contributing to the shape and the structural stability of the SARS-CoV-2 membrane and their significantly higher binding affinity is positively regulates the expression of virus-like particles [18-20]*. *The envelope (E) protein of SARS-CoV-2 contains 76 to 109 amino acids with a small (7-12 amino acids) integral hydrophilic tail in C- terminal side; whereas the transmembrane portion having a large N-terminal domain with α helical nature [21, 22] The envelope protein of SARS-CoV-2 activates viral pathogenesis through ion channel interaction [23]. The M proteins of SARS-CoV-2 range from 217 to 270 amino acid residues with three domains (membrane-spanning hydrophobic segments), a small N-terminal domain, and a large C-terminal domain are located inside and outside the virion accordingly [18, 24, 25]. Non-structural protein 3 (Nsp3) is the largest (200 kD) encoded by the SARS-CoV-2 genome which contains multiple-domains [6]. 

SARS-CoV-2 assemble promotes by spike protein which may influence by the expression of envelope and membrane proteins. Additionally D814 to G814 mutation into spike protein of SARS-CoV-2 increases the attachment with host which results enhancing infectivity [26, 27]. A Lower number of nonpolar smaller volume amino acids Ala, Gly, and a higher number of Val are observed in mesophilic proteins [28]. Psychrophilic proteins are less stable in higher temperatures containing a higher number of Phe, Try & Asp [29]. Protein stability in alkaline and the high salt environment is governed by increasing neutral hydrophilic (Arg, His, Asn, and Gln) and negatively charged (Asp and Glu) amino acid [30]. Protein stable in acidic conditions maintains a higher number of Glu and Asp on the surface [31]. Stress stable protein has favored mostly non-polar amino acids, which is the key factor for giving stability against different stresses. The proteome profile of SARS-CoV-2 was examined in this study to look at the stress stability of the protein. The SARS-CoV-2 spike (S), envelope (E), and membrane (M) proteins were also chosen to explore the same based on structural cordinates.

## MATERIALS AND METHODS

**Study design: **Our study's objective to evaluate and build up stress stability and instability of SARS-CoV-2 based on the ssRNA along with extracellular and transmembrane protein. SARS-CoV-2 Spike ssRNA Sequence were primarily obtained from GENBANK Database. Accession number of SARS-CoV-2 Wuhan: MT079854.1, Alpha: MZ314997, Beta: MZ314998.1, Delta: OK091006.1, Gama: MZ315141.1 and Omicron: OL672836.1 were retrieve for analysis. We focused on the physicochemical and structural properties of these three (the spike (S) protein, envelope (E) protein, membrane (M) protein) protein structures available on the PDB database 6CRV, 5X29 and 3I6G accordingly.

**Stability index of** **SARS-CoV-2 ss** **RNA Secondary Structures: **Vienna RNA Web Services (http://rna.tbi.univie.ac.at/) predicted the secondary structure of the RNAs of SARs-Cov-2 [32, 33]. In order to employ the viral genome sequence for analysis we retrieve it from the NCBI database. The Mfold web server (http://www.bioinfo.rpi.edu/applications/mfold/) was used to analyse the thermodynamic stability of the folding of ssRNA secondary structures [34].

**Structural Simulation with diversity: **Variant of concerns (VOCs) were reported by CDC i.e alpha, beta, gamma, delta, and omicron from the five most heavily affected countries (China, USA, India, South Africa, and Australia) also represented by “Nextstrain / ncov / open / global / 6m”. The corresponding amino acid sequences were taken and modelled [35] by using SWISS-MODEL, in order to compare the structural coordinates of the spike (S), envelope (E), membrane (M) protein of SARS-CoV-2 [35]. Using the H++ web server, the pKa value was determined for the titratable residue in the protonated state at pH 7[36]. Each protein was solvated into an explicit water box of size 10 A^0^ with a single point charge (SPC) water model TIP3P with periodic boundary condition (PBC). Each protein was modeled using OPLS3e force where Na^+^ and Cl^- ^ions were added to neutralize the charge. The system was energy minimized 2000 steps before a production run of 50 ns. RMSD and RMSF values of the backbone atoms of proteins were calculated for determining equilibrium parameters [37]. The RMSD value was calculated (calculating the average distance between the atoms) by taking the simulated structures with the Pymol software [38].

**Amino acid frequency and structural elements: **We computed the frequencies of amino acid residues in the protein sequences of the SARS-CoV-2 proteome by using MEGA [39]. Three major secondary structural regions helix, sheet and turn was determined in the extracellular and transmembrane region of the spike (S), envelope (E), membrane (M) protein of SARS-CoV-2. 

## RESULTS

**Figure 1:**
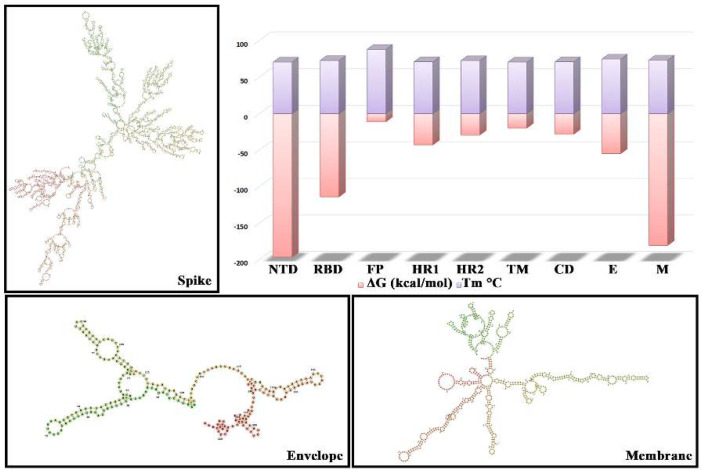
**Temperature Imprint on ssRNA Stability. **Distribution of ssRNA according to their destabilization or stabilization indexes, depending upon temperature oscillation

Based on the genomic organization, thermodynamics stability indexes of the SARS-CoV2 was analysed (Fig. 1). Virus strains are differentiated based on their genome segment and nucleotide sequence variation at individual codon positions within each genome segment. Although both strains have an identical genomic organization, they differ substantially in their nucleic acid sequences. The higher the affinity of the receptor, the stronger the binding will be between the virus and host cells. The capability of infection is the ability of the virus to enter the host cell and replicate.

The thermodynamic parameters for the SARS-CoV2 single-stranded RNA are as follows: N-terminal domain (NTD), Receptor binding domain (RBD), Fusion protein (FP), Heptapeptide Repeat 1 (HR1), Heptapeptide Repeat 2 (HR2), Transmembrane region (TM), Cytoplasmic domain (CD), Envelope (E) and Membrane (M) the melting temperature were resulted 70.5 °C, 72.7°C, 87.8°C, 70.9°C, 72.6°C, 70.5°C, 70.9°C, 74.6°C, 73.3°C; whereas the Gibbs free energy were tabulated -196.46 kcal/mol,-113.8 kcal/mol,-11.2 kcal/mol, -42.8 kcal/mol, -29.3 kcal/mol, -19.8 kcal/mol, -28.1 kcal/mol, -54.7 kcal/mol and, -180.4 kcal/mol; similarly enthalpy were studied -2011.7 kcal/mol, -1101.5 kcal/mol, -79.5 kcal/mol, -433.7 kcal/mol, -248.5 kcal/mol, -202.9 kcal/mol, -284.5 kcal/mol, -531 kcal/mol and, -1720.8 kcal/mol; the entropy were resulted -5852.7 cal/mol, -3184.5 cal/mol, -220.2 cal/mol, -1260.3 cal/mol, -822.8 cal/mol, -590.3 cal/mol, -826.6 cal/mol, -1535.7 cal/mol and, -4966.6 cal/mol. These parameters indicate that the SARS-CoV2 ssRNA is unstable accordingly (Supplimentary Table S1 and Fig. S1).

Amino acid usage patterns are influenced by natural selection pressure at the protein structural stability level; we have compared the amino acids robustness in the chosen proteome which are the most dominant factor in determining stability. Structural comparisons reveal that S, M & E of all the VOC’s are similar. RMSD value of Spike, Membrane and Envelope 0.022±0.01, 0.068 and 0.055 accordingly. Furthermore, some of the propensities of non polar amino acids (i.e. Phe, Tyr, and Gly) with polar amino acids ( i.e Thr and Ser) were higher in the case of S protein; whereas, the opposite was noticssed in E and M protein (Supplementary Table S2). 

**Figure 2:**
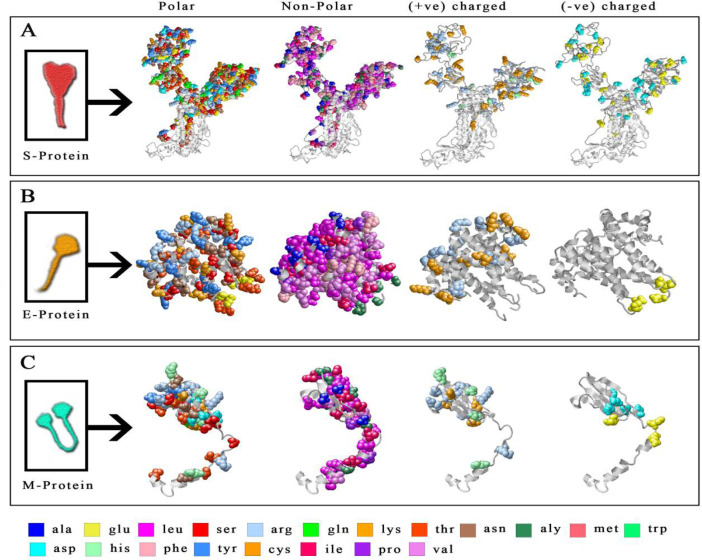
**Representation of amino acids distribution. **The amino acids were projected onto the spike (A) envelope (B) and membrane (C) protein, of SARS-CoV-2 to display polar, non-polar, positive and negative charged residues

The polar, non-polar, positive and negative charge amino acids distribution were plotted and displayed into the peptide of spike (Fig. 2A), envelope (Fig. 2B) and membrane (Fig. 2C) protein. The presence of a higher percentage (49±2) of polar amino acids in the spike protein indicates that it is more unstable against different stresses. Interestingly, the extracellular and transmembrane region of spike protein represented high polar i.e. Asn, Ser and Thr (49.9±2 %) and lesser non-polar (40.5±2%) residues. Whereas in the case of the envelope (E) and membrane (M) protein, the distribution of the non-polar amino acid is higher i.e. Gly, Ala, Val, Leu and Ile. The comparatively higher propensity of positive and negative charge amino acids was observed in Spike and Membrane protein than envelop protein of SARS-CoV-2 (Supplementary Table S2). The higher contribution of salt bridges was noted into the spike protein, which may increase stability against stresses (Fig. S2).

**Figure 3:**
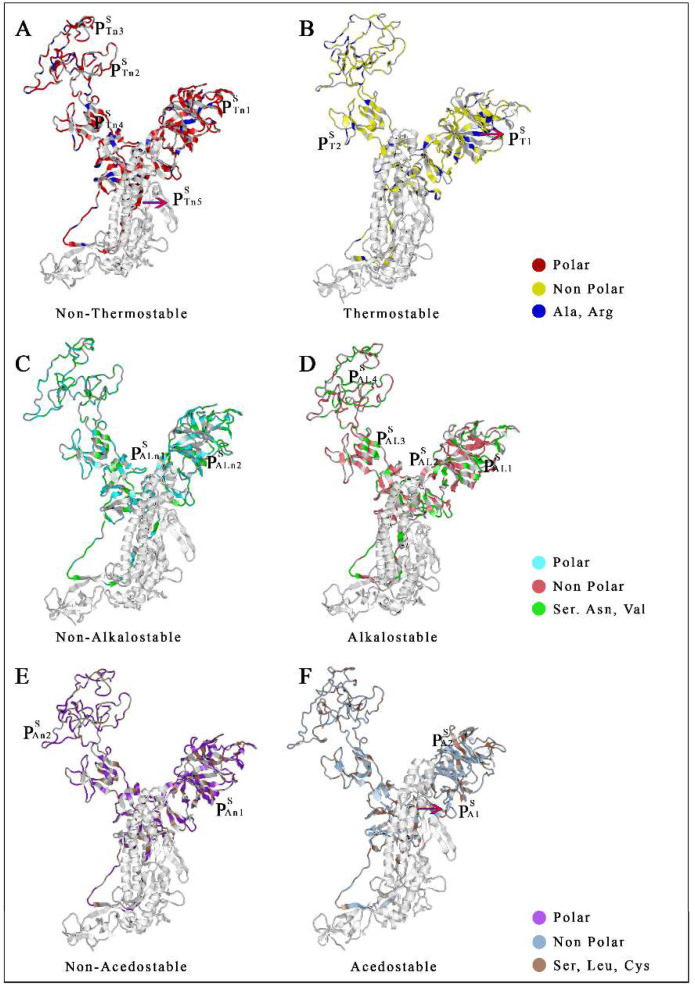
**Distribution of stress stable and unstable pockets in Spike protein. **Representation of Non-Thermostable (P^S^_Tn_) with thermostable (P^S^_T_); Non- Alkalostable (P^S^_ALn_) with Alkalostable (P^S^_AL_); Non-Acedostable (P^S^_An_) with Acedostable (P^S^_A_) pockets of the SARS-CoV-2 spike (S) protein

The spike protein interface exhibits five thermo-unstable cavity that buries residues 155, 247, 248 250, 254 and 258 in pocket1, pocket2 (residues 464, 472, 478, 484, 487, 488, 493, 516) and pocket3 (residues 369, 377, 378, 380, 385, 393, 394, 396) represented turn structure whereas pocket4 (residues 540-544,548,549,552,553) and pocket5 (residues 374, 717, 719, 724, 725, 733, 737) exhibited sheet structure (Fig. 3A). Thermo-stable pocket 1 (residues 67, 93, 243, 263, 264) displayed turn structure whereas pocket2 (residues 256,570,575) represented sheet structure (Fig. 3B). Unstable cavity against alkali condition, pocket1 (residues 53, 57, 59, 179, 273, 274, 278, 287), pocket2 (residues 63-66, 96, 170, 190, 207, 265) showed sheet structure (Fig. 3C), for alkalostable pocket1 (residues 94, 95, 125-127, 171, 264), pocket2 (residues 30, 31, 39, 60-63) and pocket3 (residues 325, 327,539, 540, 542, 551) both were sheet structure whereas pocket4 (residues 433, 437-440, 443) resulted turn structure (Fig. 3D). For non acidostable pocket1 (residues 188-191, 206, 207, 208, 214) showed sheet structure and pocket2 (residues 414, 416, 417, 420, 421, 423, 428) turn structure (Fig. 3E). For acidostable pocket1 (residues 197, 296, 298, 301, 303, 543, 605) represented helix structure and pocket2 (residues 78, 116, 117, 131, 242) sheet structure (Fig. 3F**)**.

**Figure 4:**
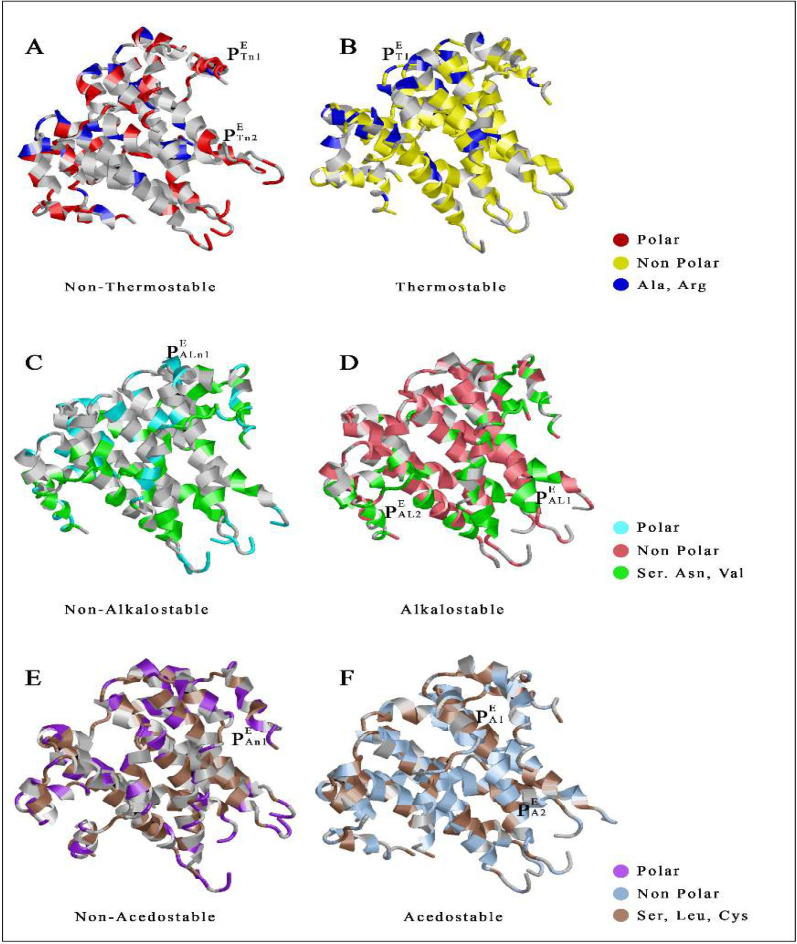
**Distribution of stress stable and unstable pockets in Envelop protein. **Representation of Non-Thermostable (P^E^_Tn_) with thermostable (P^E^_T_); Non- Alkalostable (P^E^_ALn_) with Alkalostable (P^E^_AL_); Non-Acedostable (P^E^_An_) with Acedostable (P^E^_A_) pockets of the SARS-CoV-2 envelope (E) protein

E-protein represented several stress unstable and stable complexes. The heat unstable complex, pocket1 (residues 57, 59, 60, 63) and pocket2 (residues 15, 16) both have helix structure (Fig. 4A). Whereas thermostable pocket 1 (residues 32, 36, 41) contained helix structure (Fig. 4B). In case of unstable cavity against alkaline condition, pocket1 (residues 30, 34, 38, 42,) showed helix structure (Fig. 4C). The alkalostable cluster in E protein, pocket1 (residues 15-18) and pocket2 (residues 55, 58, 60, 62) both resulted helix structure (Fig. 4D). In case of non acidostable cavity pocket1 (residues 29, 59, 61, 64) (Fig. 4E) and acidostable pocket1 (residues 25, 27, 31, 43) with acidostable pocket2 (residues 12, 16, 19, 32) displayed helix structure (Fig. 4F).

**Figure 5:**
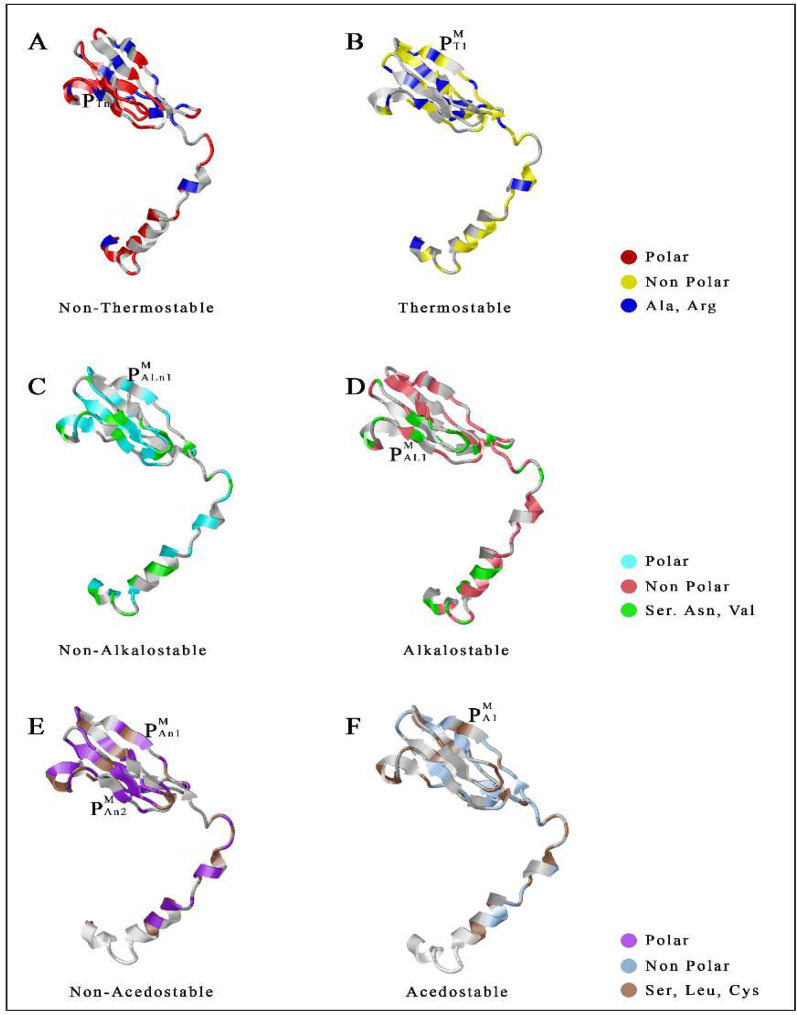
**Distribution of stress stable and unstable pockets in Membrane protein. **Representation of Non-Thermostable (P^M^_Tn_) with thermostable (P^M^_T_); Non- Alkalostable (P^M^_ALn_) with Alkalostable (P^M^_AL_); Non-Acedostable (P^M^_An_) with Acedostable (P^M^_A_) pockets of the SARS-CoV-2 membrane (M) protein

M-protein interface cavity consistently contained cavities, few of them was stress unstable with stable pockets. The heat unstable complex; pocket1 (residues 169, 179, 180, 207-209) showed sheet structure whereas pocket2 (residues 110, 116, 117, 121, 125, 199) displayed helix structure (Fig. 5A). Thermostable pocket 1 (residues 150, 158, 205, 198, 219) represented sheet structure (Fig. 5B). For non alkalostable pocket1 (residues 150, 158, 178, 179, 198, 199) (Fig. 5C) and alkalostable pocket2 (residues 170, 197, 207) both represented sheet structure (Fig. 5D). In case of non acidostable pocket1 (residues 148, 150, 158, 160, 196, 199) and pocket2 (residues 169, 183, 197, 208, 209) (Fig. 5E) along with acidostable pocket1 (residues 149, 159, 171, 197) displayed sheet structure (Fig. 5F). Different abiotic stress stable and unstable pockets of envelope and membrane proteins were identified based on the distribution and burial of amino acids. Although the stress unstable and stable cavities of were mostly contained a lower number of sheets into the extracellular and transmembrane region, compare to spike protein (Fig. S3). Our results shed new light on the specific stability index of extracellular and transmembrane protein of SARS-CoV-2 may significantly help to identify the ligand interactions specificity which may also include drugs, cell receptors and antibodies.

## DISCUSSION

Biothermodynamics is the exploration of the forces and processes that cause biological occurrence Gibbs energy is the driving force behind all natural processes. The Gibbs free energy in growth is influenced by the chemical constitution of the organism and also depends on the environment, especially intermolecular interactions between reaction participants. By taking over the host translation machinery [40], viruses have evolved a set of compact regulatory elements for successfully promoting translation and stability of their own ssRNAs. Entrance into a host, receptor binding affinity, and reproduction capacity are all chemical processes driven by changes in thermodynamic parameters, namely Gibbs energy. If the SARS-Gibbs CoV-2's energy of growth is less negative than that of its host tissue, the SARS-CoV-2 loses its pathogenicity [41].

The study aimed to find out the stress stable and unstable cavities in the membrane bound protein of SARS-CoV-2 based on amino acid distribution. Generally, it is reported that small non-enveloped viruses are mostly stable against different environmental stresses, and SARS-CoV-2 also having an envelope with its membrane [42]. The simulated atomic coordinate of each protein (S, E, M) from was used to calculate RMSD accordingly. The protein structures of SARS-CoV-2 spike, envelope, and transmembrane proteins from five countries are structurally similar; however, the spike, envelope, and transmembrane proteins from China and the United States are identical. The structural similarity of Chinese and American spike proteins may indicate perfect binding with human ACE2, resulting in a higher mortality rate. The presence of a higher number of polar amino acids indicates that spike protein of SARS-CoV-2 is not structurally stable against different abiotic stresses; although it may represent slight stability due to the presence of a significant number of salt bridges. Whereas envelope and membrane are more stable due to the higher occurrence of non-polar amino acids [29]. SARS-CoV-2 transmembrane and extracellular region of the spike protein represents five unstable (P^S^_Tn_) pockets and two stable (P^S^_T_) pockets against heat, two unstable (P^S^_ALn_) and four stable (P^S^_AL_) pockets against alkali, two unstable (P^S^_An_) and stable (P^S^_A_) pockets against acidic conditions (Fig. 3). As the spike protein induces attachment of virus membrane with the host cell membrane, so instability indexes are to be used to dissociate human ACE2 and SARS-CoV-2 spike protein interaction. In the case of the envelope protein, transmembrane and the extracellular region displayed two unstable (P^E^_Tn_) pockets and one stable (P^E^_T_) pocket against heat, one unstable (P^E^_ALn_; P^E^_An_) and two stable (P^E^_AL_; P^E^_A_) pockets against alkali with an acidic condition (Figure 4). Similarly, M protein was displayed two unstable (P^M^_Tn_; P^M^_An_) pockets and one stable (P^M^_T_; P^M^_A_) pocket against heat and acidic conditions; similarly it also represented one unstable (P^M^_ALn_) and stable (P^M^_AL_) pocket against alkaline conditions (Figure 5). SARS-CoV-2 is highly susceptible in a favorable environment; it is highly stable at 4°C but unstable above 39°C [43, 44]. Hydrophobic tails of lipid bilayer interact with alkali molecules which destroy the membrane architecture of viruses [45, 46]. 

Spike protein of SARS-CoV-2 binds significantly to human ACE2, TMPRSS2 and CD26 are already reported through several amino acid residual interactions. We found a heat unstable pocket (P^S^Tn2) in the interaction site of human ACE2 with SARS-CoV-2 spike protein (Supplementary Table S3), which may be considered for further studies for the development of a new efficient anti-corona drug [47, 48]. The stress unstable pockets in spike (S), envelope (E) and membrane (M) protein also represent several epitopic regions in it (Supplementary Table S3-S5). The association with the epitopes with these pockets may be an important clue for the selection of effective epitopic signature for future antibody production against SARS-CoV-2 [49, 50]. Our results demonstrate for screening the rapid discovery of novel inhibitor molecules depending on the stable and unstable footprint located on or into the membrane associate proteins.

## Acknowledgements:

We thank the Oriental Institute of Science and Technology, India for supporting our work.

## Conflict of Interest:

The authors declare that they have no conflict of interest.

## Supplementary Materials

Supplementary Material 1

Supplementary Material 2
