# Supplementary material for: Stress adaptation signature into the functional units of spike, envelope, membrane protein and ssRNA of SARS-CoV-2
Source: Mol Biol Res Commun. 2022;11(4):155–66. doi: 10.22099/mbrc.2022.44594.1777 (PMC9905751; doi:10.22099/mbrc.2022.44594.1777)
Supplement: Supplementary Material 2 [file mbrc-11-155-s2.pdf]

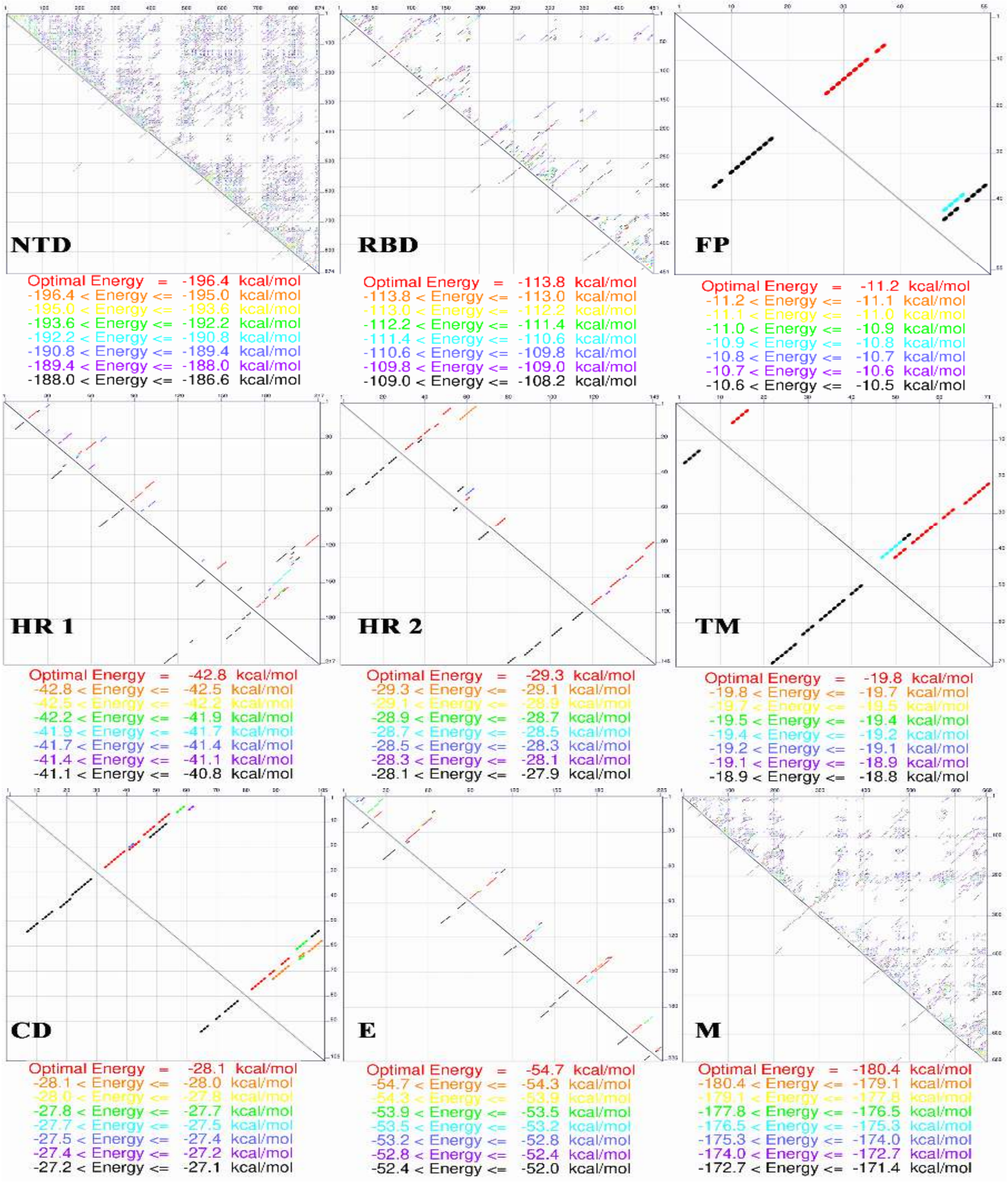

Figure S1: Comparison of Gibbs energies of formation and stability

The reaction quotient's impact on the increase of Gibbs free energy. The impacts of the common Gibbs energy of growth are explored in the figure about of different functional region of ssRNA of SARS-CoV-2 N-terminal domain (NTD), Receptor binding domain (RBD), Fusion protein (FP), Heptapeptide Repeat 1(HR1), Heptapeptide Repeat 2(HR2), Transmembrane region (TM), Cytoplasmic domain (CD), Envelope (E) and Membrane (M).

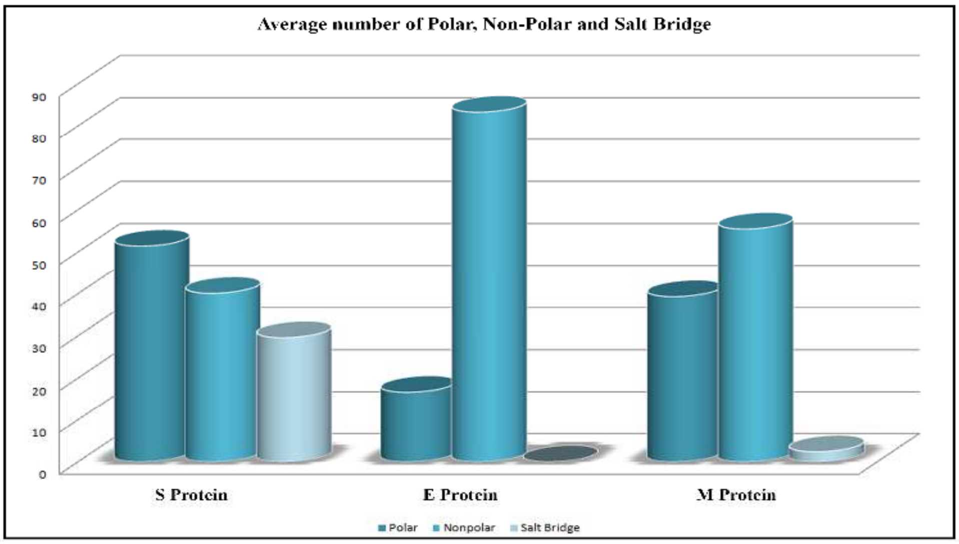

Figure S2: Occurrence of polar amino acid, non-polar amino acid and salt bridges in Extracellular and Transmembrane region

The average number of polar amino acid, non-polar amino acid and Salt bridges is plotted against the different SARS-CoV-2 strains isolated from China in favour of the spike (S), membrane (M) and envelope (E) protein.

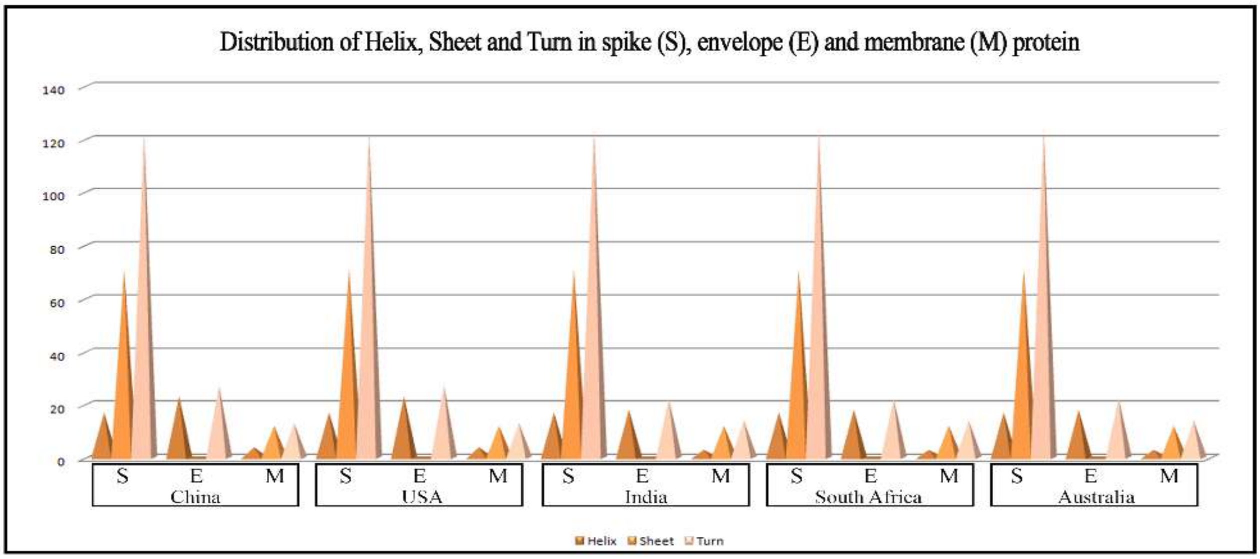

Figure S3: Secondary structural components in spike, membrane and envelope protein

Structural summary of refinement secondary structure elements (Helix, Sheet and Turn) distribution in a different context of SARS-CoV-2 strains isolated from China considering spike (S, membrane (M) and envelope (E) protein.
